# Introductory Lectures

**Published:** 1848-03

**Authors:** 


// BUFFALO MEDICAL JOURNAL

AND

MONTHLY REVIEW.

VOL. 3. MARCH, 1848. NO. 10.

### ORIGINAL COMMUNICATIONS.

---

ART. I.—*Introductory Lectures.*—Addresses delivered at the opening of the lecture terms at Medical Institutions in different parts of the country, constitute an important branch of our medical periodical literature. It has become an established custom at most schools, for one, or more of these introductory discourses to be prepared with a view to publication, or, if not so prepared (as is sometimes modestly disclaimed) the writers are induced to yield to the solicitations of their hearers, and consent to see themselves in print. For ourselves we like the custom, and hope it will not pass out of vogue until all the topics appropriate for such an occasion are as stale, flat, and unprofitable, as the subjects of fourth of July orations have, at length, become. At the present time, when the minds of the profession are earnestly engaged with matters of common concern, and of not a little importance, relating to reform, progress, education, &c., these addresses possess an especial interest. As a general remark they are to be taken as exponents of the views, not of their authors only, but of the different Institutions from which they emanate. We are thus enabled to compare the sentiments, not only of a few individuals, but of the different sectional associations which these individuals may be supposed to represent, and to gather expressions of opinion on debatable topics from widely separated districts.

By means of these addresses we are able to form some opinion of the condition, aims, and prospects of the numerous medical Institutions of our country, and while doubtless they are often designed to advise the profession of the successful operation of the institutions with which their authors are connected, they are not the less valuable as containing information in which every friend of medical education should feel an interest.

They are acceptable, also, inasmuch as they carry us back to the period when we were hearers, not readers of such discourses—a period which we learn to prize when it is gone, forever gone! In imagination the wheels of time roll backward, and we are transported to that epoch in our own experience when the long expected occasion of meeting our teachers and fellow pupils has arrived. The thoughts, feelings, and associations of the medical student, are, for the moment, brought vividly before us. We live again that portion of life's history which was rendered happy by bright dreams of the future—alas! how little did we then imagine that one of the highest pleasures of that future, would consist in living again in the past!

There is another point of view in which these annual missives are acceptable. We seem to form a personal acquaintance with the authors, of most of whom we often hear, but with whom we may never come into nearer relation than through this intellectual medium. In many instances these are the only occasions on which members of our profession who are eminent as teachers, afford an opportunity for distant brethren to become acquainted with them even by means of their writings.

Not to multiply reasons, we repeat, that in our estimation the custom of publishing introductories is a good custom, and we hope it will become time-honored ere it wear itself out, and, from the dearth of novelty, become extinct.

The harvest of introductories for the present season appears to have been as abundant as usual, and we are so fortunate as to come in for a fair share of the products. The authors of published addresses at several schools have kindly remembered us, for which we embrace this opportunity to tender our thanks. We should have expressed acknowledgments ere this, and for each favor separately, but we did not deem it just to the authors to speak of their works until we had read them, and as our engagements have compelled us to defer that gratification in several instances, we preferred to file away these publications by themselves, and to devote an early period of leisure to them exclusively, reading them in succession until our stock was exhausted, In this way we promised ourselves the enjoyment of a bountiful repast made up of a variety of dishes, instead of tit-bits from time to time, which, although sufficiently savory and wholesome, are better calculated to provoke appetite, than to satisfy hunger. We have now placed the budget on our editorial table, and purpose as we take up successively each discourse, to present to our readers extracts possessing intrinsic interest, or which shall seem to us to be fair specimens of the merits of the production. Presuming that the major portion of our readers have not the opportunity of reading all the discourses before us in full, we have thought the space which our selections will require could not be more acceptably occupied. In noticing each discourse we shall exercise the privilege of making such critical remarks as suggest themselves during our perusal of it.

In looking over the list, we select, first, those which bear the imprint of Philadelphia; and of these, two discourses by the Professor of Surgery in the University of Pennsylvania first meet the eye. They are entitled as follows:—one, "*lecture, introductory to a course, on Surgery, containing a short account of eminent British Surgeons, Physicians, Scientific and literary men;*" the other, "*lecture, correlative to a course, on Surgery, embracing a short acccunt of eminent Belgian Surgeons, Physicians, &c., &c.*" The materials for both lectures were gleaned during an European tour made the summer preceding their delivery, and relate, for the most part, to eminent members of the profession with whom the lecturer was thrown in social intercourse, or on public occasions. We select first an account of a lecture delivered by Dr. Carpenter which we think our readers will peruse with interest. Having given a brief but glowing account of a lecture by Faraday, the eminent chemist, he proceeds as follows:—

"The next individual towards whom I was attracted at the association, both on account of his high reputation, and the acquaintance I had made with him in former years, was Dr. Carpenter, recently of Bristol, now of London, where he occupies the important positions of lecturer on physiology at the London Hospital, examiner on physiology and comparative anatomy in the University of London, lecturer on geology to the British Museum, a lectureship recently created by the will of the eccentric founder, the late Dr. Sweeny. Dr. Carpenter, in addition to these honorable apointments, is also Fullerian Professor of Physiology in the Royal Institution, where he has just completed a three years' course of lectures on that branch of science. This professorship expires every third year, and it is probable he will be elected again to the same office. When it is considered that Dr. Carpenter has held high rank as a writer for the last ten years, and published his valuable work on physiology, *whilst a student;* that he is now only about thirty-one years of age, and has created for himself, within that short period, an enviable reputation in every portion of the civilized world, it may be readily imagined that in meeting him at the British Association, I should feel disposed to devote more than ordinary attention to any subject which might happen to come under his investigation. Finding, accordingly, that he was to deliver a lecture on the physiology of the encephalon, I determined to devote myself assiduously to the physiological section, until I could make myself master of his views. He commenced by reference to a lecture delivered before the same association in 1846, and gave a brief recapitulation of the views then stated, from the published reports of that body. The object of that communication was to bring under consideration the inferences to which we are led by the study of comparative anatomy, in regard to the functions of different parts of the human encephalon. The author considered that the great principle of Cuvier—that the different classes of animals might be regarded as so many experiments ready prepared for us by nature, who adds or takes from their various organs, and shows us the several results of these different combinations—though fully recognized in other departments of physiology, had not yet been fairly admitted into the study of the nervous system. He attributed this, in part, to the difficulty of determining the portion of that system which are truely analogous, or rather *homologous*, in different classes of animals; but this difficulty has, in great part, been removed by the growing conformity of opinion on this subject amongst comparative anatomists, so that he thought that the time was come for physiological deduction.

He first pointed out that our comparisons need not be restricted to vertebrated animals, since the ganglionic centres of invertebrata may be shown to bo analogous to certain portions of the cerebrospinal system of the vertebrata. He stated it to be a universal fact, that all organs of special sense have distinct ganglionic centres, which must be regarded as the instruments of their respective sensations, and as the sources of motion directly connected with those sensations; and that the whole cephalic mass of invertebrated animals was composed of a collection of such ganglia, without any vestige—except in the highest—of cerebrum or cerebellum. These organs make their first appearance in fishes, and bear, at first, but a small proportion to the chain of sensory ganglia which forms the anterior termination of the spinal cord.

In fishes we find distinct olfactive, optic and auditory nervous ganglia, together with thalami optici and corpora striata, the degree of development of which has no reference to that of the cerebrum; in fact, the bodies usually called the cerebral lobes of fishes, are, except in the sharks, which have vestiges of cerebral hemispheres, almost entirely composed of the homologues of the corpora striata. Hence Dr. Carpenter considered that these bodies, instead of being appendages to the cerebrum, really belong to the group of sensorial ganglia, and are to be regarded as together making up the ganglionic centres of common or tactile sensation, and of the movements prompted or directed by it. This chain of ganglia, although comparatively small in man, with reference to the bulk of the cerebral hemispheres, still exists in him; and must be regarded as the instruments of the same operations as those to which it ministers in the lower animals. Arguing from actions in the latter, and analogous phenomena in man in health and in disease, the author attributes to the sensory ganglia the formation of sensations and the origination of respondent movements. To this category the purely instinctive actions of the lower animals, which seem executed without any *idea* of purpose, but in simple respondence to the promptings of sensation, appear referable; together with a variety of actions in man; such as that of yawning from the sight or sound of the act in another. Dr. Carpenter hence endeavored to show that we must regard the cerebrum as the instrument of the formation of ideas, of the memory of *ideas* and sensations, and of the intellectual processes founded upon them, which terminate in an act of the will; and he pointed out that *ideas* may produce the same effect on muscular movements as sensations themselves, as when the suggestion of the idea of yawning induces the action. He also showed how the anatomical connections of the cerebrum with the sensory ganglia would cause its communicating fibres to exert an influence on the latter, corresponding with that which is effected by the sensations directly received from the organs of sense. With respect to the emotions, he endeavored to show that they may be regarded as compound states resulting from the simple feelings of pleasure and pain associated with certain ideas, or classes of ideas; the feelings of pleasure or pain he would locate with the sensations which commonly excite them in the sensorial ganglia; whilst the formation of the ideas, which are essential parts of the motives and propensities, is clearly a cerebral operation; and he showed, in conclusion, how this view of the functions of the principal parts of the encephalon harmonizes with the known sleepless action of the emotions—first, in producing involuntary movements, and secondly, in stimulating and influencing the reasoning processes.

After a brief recapitulation of the preceding deductions from comparative anatomy, Dr. Carpenter brought under the notice of the medical section two remarkable cases, which he considered to be peculiarly illustrative and confirmatory of his views. In one of these communicated to him by Dr. Dunn, all harmonized well with the supposition that the functional power of the cerebrum and of the auditory, olfactive and gustatory ganglia, was suspended, whilst that of the optic and tactile ganglia remained in full force. The actions of the individual, for a long period, were such as might be legitimately referred to the *consensual* group; being all stimulated by present sensations, and not being, apparently, in the least degree the result of *will*, guided by *intelligence*. In the other case communicated by Mr. Noble, the intelligence and will were in full operation, but from a defect in the power of the will to excite muscular movement, the individual could not exert his volition, unless the object of the exertion were before his senses. Thus he stated that if he were assured that upon extending his hand to a given point, he should receive a one hundred pound note, he could not make the movement unless the note were before his eyes; the sight of it thus guiding the action *consensually*."

The following lively account of his interviews with the late Mr. Liston exibits another phase of the writer's style:—

"Few hours elapsed, after reaching the metropolis, before I found my way to the domicile of my old friend *Liston*. I was shown into a pill-box of a room, tastefully fitted up, and filled with medical and surgical portraits, from old Abernethy, with his hands knowingly thrust into his breeches pocket, down to Jobert of Paris, with his enormous black whiskers and heroic figure and phiz. Whilst intently gazing upon these, the door noiselessly opened behind me, and a hand laid heavily enough upon my shoulder to face me about, was followed by the exclamation, "what under the sun has brought you here again?" Before I could answer the question, I was hurried into another room, and both sinking simultaneously into luxurous arm-chairs, a conversation was commenced and continued for a considerable time, occasionally interrupted by the pressure of the sides and whisking of the tail of the enormous black cat Tobey upon almost every part of my person,—this respectable gentleman, now grown corpulent, no doubt recollecting the libel I had published in relation to his foot and my plate of soup, and above all in bestowing upon him the common epithet of Tom, instead of the more honorable and dignified one of Tobey. Upon taking leave, Liston said to me, "Come here at eight o'clock to-morrow morning, and I'll take you down the Thames in my yacht, and then out to sea, and show you fun you never dreamed of on board your splendid packet-ships." I excused myself upon the ground of having made a previous engagement with Sir Benjamin Brodie and Dr. Bright—inwardly pleased at the opportunity of escaping all the *de sagremens* of sea-sickness, which is sure to follow an excursion in such a craft, with a positive certainty of being visited upon any one even like myself, who had crossed the Atlantic numerous times without being subjected to any sickness at all. A few days after this I saw my friend in all his glory at the North London Hospital, his lofty forehead surmounted by a cap, his broad chest and figure covered in part with an apron, and his right arm brandishing a long and dagger-shaped knife. I came upon him unawares and stuck a finger into his side, when he immediately turned round, and said to the fine-looking young men who composed his class, "Ah, this is the American professor who has libeled my cat by calling him Tom." He then turned round, introduced me to Professors Thompson, Quain and Grant, gave me a position where I could distinctly see all he was going to do, and with a graceful turn of the body and corresponding sweep of the knife, upon a patient previously etherized and prepared for the operation, speedily worked out a pair of anterior and posterior flaps, and almost in the twinkling of an eye amputated the limb as near to the hip as he thought it proper and safe to go—the poor girl not giving the slightest demonstration, by movement or scream, of what had been done. Some minor operations followed—all performed in the same quiet, easy, unpretending way, without any flourish of trumpets or display of flashing blades, and almost without the patients and by-standers being aware that so much could be effected with so little formality and fuss. Soon after this I took leave of Liston and left for Paris. Upon my return in September, I called again, and learned from him with regret that he had been ill soon after I had parted from him, and had lost thirty ounces of blood from an abscess in the throat, brought on, as he imagined, from attendance upon medical friends laboring under malignant erysipelatous disease. To recover from this severe attack, fortunately unconnected with the trachea or lungs, he had gone to Scotland among his friends, and after freedom from exertion and care, by reveling amidst the romantic scenery of his native hills had returned to London in better health than he had enjoyed for many years. Before leaving London I saw him repeatedly, dined with him and Tobey and other friends in the most unrestrained way, talked on all subjects, professional, grave, farcical and funny, heard many queer anecdotes and histories of men, and hospitals, and medical schools, went back to the days when *he* was reveling at Edinburgh in the sweets of Barclay's rooms, and *I* in those of Monro—neither of us dreaming at the time that we should ever occupy similar chairs, or preach to those who some twenty years hence will be destined to play a part in the same drama, where they are now merely scene-shifters behind the stage, and who, in turn, will tell *their* pupils many old stories about their masters and contemporaries in days of "auld lang syne." "Sic unda supervenit undam," and it is only right and just that it should be so."

The abscess in the throat alluded to in the foregoing extract, was an aneurism, which shortly afterward proved fatal. The character of the disease, it seems, was not ascertained previous to his decease.

Passing to the second lecture, we give his account of Dr. Suetin, of Brussels, inventor of a modification of the *immoveable apparatus* called the *Bandage Amidonne.*

"The next day, at the appointed hour, Parkinson and myself directed our steps to the *Hopital St. Pierre*, and were soon joined by our friend Clemson, as an *amateur*. Half an hour elapsed, without evidence af Suetin, and his roistering car. being near. At last he came, and, out of breath, bolted through the shady grove, surrounding the venerable pile of buildings, crowded with patients of every age and sex—all too happy, when allowed to escape from its old and darksome wards, and to promenade, or rest, beneath the boughs of wide-spread beech and towering elms, where we ourselves had sought a cool and pleasant retreat from an oppressive morning sun. As he approached the spot where we stood, he joyously cried out: "There, you see, at once, how much can be done by my bandages, starch and cartons. Look at those boys with broken thighs and legs; see how they are skipping about with crutches and sticks, and some of their fractures not twelve days old! Could they do as much if encumbered by the bandages and splints of Boyer and Desault?" We were not prepared to gainsay his statements, or dispute his facts, which seemed to stare us in the face, and say—"I dare you to deny what he has advanced." "And now," he continued, "let me go to work, and give you further proofs of the excellence of my plans; for, although I have no perfectly recent case in the house upon which to demonstrate my methods and views, I will take, as an example, that boy's thigh, fractured eleven days ago, remove the dressings, and let you see for yourselves, whether it is shortened or, in any way deformed." Accordingly he commenced with a pair of strong spring shears, sharp at the extremity of one blade, probe-pointed at the other, to nip, or cut through the hardened case, which inclosed the limb, to the extent of a quarter of an inch at a time, continuing to cut until he had split the covering from the foot to the hip. Then, applying his fingers to the edges of the cut case, he *carefully* separated them from the limb by slow and gradual eversion: taking *especial* pains to support the thigh at the fractured part, and to disturb the whole limb as little as possible, until he effected its complete delivery from the mould, in which it had, as it were, been cast.

Its appearance, as he predicted, indicating nothing unfavorable, either on the surface of the skin, or in the position of the fragments of bone, he next placed, longitudinally, some *linen* strips, or compresses, over the skin, especially about the seat of fracture; then took a narrow roller, made of old, thick, but very soft, flexible *linen*, and applied it circularly, and with very moderate firmness, to the whole limb, with exception of the instep and toes, which were left uncovered as an index of the condition of the inclosed parts. A painter's brush, dipped in common starch, was then applied to the *edges* of the roller, and not over its entire surface, merely to connect them together externally: and, thereby, to prevent it from becoming stiff, and communicating close and disagreeable pressure to the skin beneath. Over the roller, thus applied, were laid parellel with the limb, and throughout its whole extent, several bags, or junks, about an inch and a half wide and half an inch thick, filled with tow: and between these, and also parallel with them, a piece of tape, the ends of which extended beyond the toes and hip. Another roller was made to surround the limb and junks, and starch applied to its edges, in the same cautious way. Over this second roller were then placed thick paper-splints, carton, or binder's boards, light, spongy, and soft, and free from glue, or any similar material calculated to stiffen them. These splints, previously softened by water extended from the spine of the ilium to the toes, were hollowed out and moulded to the limb, so as to surround it completely, and form a base, with the exception of the instep and toes. While these were held in their respective situations by assistants, Suetin applied another roller, well starched, except that portion which covered the *ridge of tha tibia*, and connected all the splints firmly together. His object in keeping the starch from touching the dressings over the tibia, was to avoid irritation, which he had from, experience, found uncomfortable to patients, and injurious. Shorter bands, or rollers, were next applied, circularly, especially over the seat of fracture, and well plastered with starch. A loop, made out of strong linen, was also connected by starch and rollers to the foot, and hung beneath its sole, for the purpose of extension, in case of shortening of the limb, by overlapping of the fragments. To fill up inequalities, and to prevent the starched edges of the rollers from giving pain, by their hardness, layers of carded cotton were placed in various situations, previous to the application of the splints. The whole process was completed by placing a piece of oiled silk, seven or eight inches square, on the inner and upper part of the thigh, to prevent the dressings from being soaked by urine, and the excoriation consequent thereto. In several other cases similar dressings were applied by Suetin—only modified by peculiarities of each case, and the individual bones broken, and in all with the same favorable result—without pain or complaint on the part of the patients, and with every prospect of perfect cure, whilst walking about with the assistance of a stick, or crutch. Under progress of treatment were shown to us, upon the same occasion, ten or twelve cases of fractured thighs, legs, arms, clavicles, all in a fair way towards consolidation of fragments and cure, without confinement to bed, or to the hospital, except at night. In the calvicle cases, the apparatus of Desault was applied, and the outer bandages, being well brushed and saturated with starch, seemed to hold their places so firmly as not to require, probably, a removal until a cure should be effected. After showing us all the wards of this large and ancient hospital, prescribing for its numerous syphilitic patients, male and female, and for various cases of medical surgery, *Suetin's* attention was called to one of those obscure and complicated fractures of the radius, just above the wrist, which the most eminent surgeons have found so difficult to detect and treat. The injury had been sustained a few hours before; and swelling having supervened, it became extremely difficult for any one to pronounce decisively, respecting the exact nature of the injury. Fatigued as he was, however, and almost worn out by the exertions he had made for several hours, in demonstrating, for our benefit, the principles which governed him, and the practice he employed in the treatment, not only of fractures, but of various other diseases, such as abscesses, ulcerations, carious joints, coxalgia, varicose veins, by the "*Bandage Amidonne*," he repaired, at once, to the assistance of the patient, examined his wrist, pronounced the injury to be a fracture of the radius, half an inch above its lower extremity, applied his dressings in such a way as to preserve the *interosseous space*, controlled the hand by a broad splint, extending beyond the fingers, fastened all together by his starched rollers, placed the forearm in a flexed position, sustained it by a sling, and promised the patient a perfect cure in two or three weeks. "How long," said I, "will you keep that apparatus applied?" "Only for two or three days." was the reply, "If." he continued, "it were suffered to remain too long, and without passive motion of the wrist, anchylosis or stiffness of tendons, might ensue." This convinced me he knew what he was about, and I left the hospital much edified by all I had seen; and convinced that the immovable apparatus, if managed with the skill and caution of a *Suetin*, is decidedly superior, in the generality of cases, to most other dressings; and equally well calculated to inflict a vast amount of injury, if employed by those ignorant of its mechanism and power."

This lecture closes with a narrative of the love, crime, and penitence of *Versalius*, the anatomist, which is said to have peen lately discovered written on parchment enclosed between two paintings by Titian, and purports to have been written by Versalius himself. The story has no relations to medicine except that the hero and autobiograoher was an anatomist, yet such is its romantic, touching character, that the reader will not complain that it occupies about one third of the discourse. Were it more brief we should be tempted to transfer it to our columns.

If it were considered requisite that the subjects treated of in introductory discourses should have, at least, some remote connection with, or reference to those which are subsequently to be presented to the student in the course to which the discourse is introductory, the lectures of Prof. Gibson would be, on this score, obnoxious to criticism. Custom, however, appears to sanction the widest divergence from any such relation. The occasion is one on which the lecturer is at liberty to select any theme which suits his fancy. Whether there is an intrinsic propriety in this we do not say; but conventionality has established it, and we have therefore no right to quarrel with Prof. G. for availing himself of the privilege. Putting relevancy of topics out of the question, it may be doubted if these lectures are in perfect keeping with the time and place of their delivery. A captious critic might be disposed to ridicule an appearance of vanity, which leads the author in all his portraitures to represent himself as not the least prominent character in the picture. But it would be ill-natured, if not ungrateful, to take exceptions of this sort, when we are bound to confess that of all the performances of a similar character before us, there is perhaps not one which will be found to contain more entertaining, and useful information. Egotism on such an occasion is certainly less objectionable than on some other occasions; and it is far more excusable in Prof. G. than it would be in one less deservedly eminent.

The Jefferson Medical College has been unusually prolific in introductories the present session. Addresses by Professors Dunglison, Mitchell and Mutter, were published by the class.

Prof. Dunglison selects as the topics of his discourse some of the superstitious notions, and delusions, which appertain to the origin and history of medicine. We give the following extract from the concluding portion of the address:—

"It may be inferred, then, that from every passing sect or system; from every curative observance, rational or empirical, the judicious therapeutist may extract something that may tend to the advancement of the science, and the extension of his sphere of usefulness; and that it is the duty—as it ought to be the pleasure—of the philanthropic physician to adopt every improvement, no matter from what source it may emanate. To attempt, by the active opposition of the profession, to arrest quackery, even of the most contemptible kind, in its career, would be futile:—nay, more, the opposition is apt to be regarded by the laity as persecution, and persecution suggested by interested and sordid considerations. The human mind, moreover, is so constituted, that it loves mystery; and reposes more faith in that which is hidden—if unblushingly proclaimed—than in the most open and candid exhibition of really potent agents. All that is left, therefore, is for the profession, in every case, to wait until the mystery has been removed, and then to endeavor to appreciate and embrace the good that may flow from it.

Such is the view which reason and philanthropy would compel us to adopt; and if there be any thing perhaps that distinguishes the present condition of the medical mind from the past, it is the disposition of the wisest members of the profession to admit those principles more as rules of action than they did formerly. It is easier, however, to observe than to think; and hence the number of most useful, but not necessarily scientific, pioneers of science, who restrict their attention to contemplating and classifying morbid specimens as they would objects of natural history; without reflecting in what manner they may lead to the saving of life or the alleviation of suffering,—without seeming to give a thought, indeed to those all important results. With many of such individuals the disposition has been to narrow down the science of medicine to the mere observation of facts:—to carry it back to those periods when the ancient empirics "saw without discerning, administered without discriminating, and concluded without reasoning,"—a disposition which has been fostered by a recent writer of our own country in an able essay on what he terms the "Philosophy of Medical Science," who—after deprecating in place of lauding, as he ought to do—"the almost universal mania"—to use his own language—"which exists for explanations and interpretations of all phenomena"—remarks:

"The dominant feeling in the American medical mind, seems to be,—not what *are* the facts and their relationships—to what extent and with what degree of positiveness and accuracy, have they been ascertained?—but *why* are these facts and relationships such as is alleged? And *how* are they so? A vastly greater degree of importance is often attached to the possible, though, perhaps, wholly unattainable *why*, and *how*, and *wherefore* of the phenomena, than to the phenomena themselves; and in strict conformity to the requisitions of this strange philosophy, in many cases, unless some plausible or satisfactory answer can be given to these questions, the very existence of the phenomena themselves is coolly and complacently denied! We have practically reversed one of the sayings of John Hunter—'Don't think, but try'—and adopted its opposite—'Don't try, but think.'"

Yet John Hunter—possessed of the very highest qualifications as an investigator of nature — "unwearied"—as he has been described—"in induction, sagacious in grouping together analogous phenomena, and ever striving to ascend from propositions of less to those of greater generality," could scarcely have given utterance to so unphilosophical a sentiment. To try without thinking would be the veriest empiricism. It could, at best, lead to an additional accumulation of the so-called facts—some true, others false—which have been piled upon each other in glorious confusion for ages, and have ever weighed on the science;—from which we are daily, indeed, endeavoring to free ourselves. The mere observer of phenomena, who thinks not, is but the unenviable counterpart of him, who

> ——"Saw with his own eyes the moon was round,
> Was also certain that the earth was square;
> Because he had journey'd fifty miles, and found
> No sign that it was circular anywhere."

The true philosopher, who wishes to interrogate nature on any branch of science, must, first of all, reflect or form his hypothesis; think, in other words, and then try. If he finds the results indicate that the phenomena do not accord with the hypothesis, and that it is insufficient, he abandons it, and has recourse to another—equally suggested by thinking—until ultimately he succeeds in adding one more step to the ladder of science. Thought suggests the experiment; experiment leads to observation, and thought again fixes the relationship. No number of facts or phenomena can constitute a science. The science—as before remarked—does not exist until we have systematized and deduced the laws of such phenomena. The maxim ascribed to Hunter ought, therefore, to be in reality reversed; and the one substituted for it should be:—"*Think* AND *try*." To enable you to do this satisfactorily will be the lofty object which I shall have in view throughout the course that it now opening before us. It will be my endeavor to sift for you the true from the false; the hypothetical assumption from the demonstrated law; to render you familiar with the phenomena of the science—the results of observations—but still more, to familiarize you with the investigation of the laws of these phenomena; to furnish you—in other words—with materials for thinking, and to render the process more easy to you."

Dr. Mitchell's address is an interesting and able performance, although desultory. He touches upon various points relating to education, and the condition of the profession of this country, from which we might select texts which would lead to extended comments. We will quote some passages upon different topics, adding, however, but few annotations. Speaking of private medical instruction, he says:—

"The system of instruction is imperfect, and must ever be so to students collected in classes. It is only in the office, and under the care of his private instructor, that the learner can reach the full benefit of practical education, or be thoroughly fitted to commence the practice of medicine. What the dissecting room is to the anatomist, and the dinner-table to the carver, are the lessons of the private preceptor to the medical freshman. With him, he can listen to the pulmonary motions, apply his ear to the cardiac region, and explore by palpation, the size and hardness of the liver, and thus obtain a practical persuasion of medical truth, which all the lecturing in the world cannot convey. It is in this respect that the education of physicians is most faulty, and I had hoped that the learned and dignified Convention, which met, in May, in Philadelphia, would have resolved to render more perfect this most essential part of the education of physicians."

For these, and other advantages to be derived from private instruction the writer enforces the necessity of *long vacations* between terms of lectures, from which we infer he is not in favor of extending the period of the latter to six months. The drift of a considerable portion of the discourse seems to be with reference to that point.

The following is apparently designed as a hit of the National Convention and those institutions which have adopted its recommendations:—

"There is at present, in all institutions, a kind of insurrectionary spirit for alteration and amendment. I say insurrectionary, because the spirit exists, not only where reform is really needed, but where alteration would do harm. The love of change has come over the people, and in morals, religion, temperance, anti-masonry, anti-slavery, fourierism, and nativeism, efforts after new conditions are on the wings of a vigorous enterprise. It was hardly to be wondered at, that medicine should catch at last the rank infection, and that physicians should also long for change, and endeavor to govern on some inflexible system, the schools and the legislation of nearly thirty states· But they have caught it, and have bestirred themselves to make things mend faster than would the general progress of society. The motive is good. The honor and interest of the first of professions are worthy of all the time and trouble bestowed upon it. But experiments must be made cautiously, The complex relations of medicine to society, require careful thought and long deliberation. And after all our care, too meddling a legislation has ever, by clipping too closely the wings of enterprise, endangered its safety, whilst it impeded its swiftness·"

The sentiments contained in the following quotation are as just, as they are patriotic. We commend to the attention of the reader the concluding paragraph:—

"Ever since the settlement of America, the condition of every thing in this country has been the constant theme of abuse. A wholesale dealer in slander, the great naturalist Buffon, denounced our climate and soil as scarcely fit for the habitation of man, and asserted that everything was belittled by them; that animals diminished in size, activity and courage, and that man, too, would degenerate into an enervated and useless being. This opinion, adopted by all Europe, without examination, was reiterated by the travellers in our own country so often, as to pass for an established truism on both sides of the Atlantic. The local prejudice which ties all other people with links of affectionate adamant to the soil upon which they were born, seemed wanting in the American, and with the humility of a provincial and a colonist, he looked to the mother country as the pattern of all excellence. No one deemed it worth the trouble to examine the foundation of such opinions, and they passed into unquestioned currency. An American was an inferior animal, his institutions were absurd, his climate destructive. Religion and morals were alleged to be here at their lowest ebb, and an American book of ordinary cleverness was esteemed a curiosity. Our noble profession came in for its full share of abuse, and the detraction of the last age yet hangs about it, an hereditary impediment. This delusion is sustained chiefly by those, who have, with a laudable desire for improvement, resorted to the foreign shools of medicine; but who, instead of sitting in judgment on the systems and men of Europe, implicitly adopt their authority, and return to despise the home and the profession which, as a whole, I firmly believe to have no equal.

In continuation of the same train of remark we quote the concluding portion of the address:—

"Enough has been adduced to show that we are not to draw our rules from the conservative wisdom of the old world, for any American purpose. Our institutions, our climate, our people are peculiar, and peculiarly as a nation are we situated. Ours is a land of progress; one in which men are to reach their ends by new and untried paths. The slow movement of European systems suits us not, and we must look hereafter with more distrust on the men of foreign or of domestic growth, who see in us nothing good, and believe that we can only succeed in policy, war or medicine, by a blind subserviency to orders from abroad.

After all the abuse that we have ourselves lately heaped upon American medicine, by way of convincing the public that it ought to distrust us, we loudly complain that it takes us at our word, and flies to homœpathy and hydropathy, whose practitioners do not expose their own absurdities. This conduct is the more inexcusable, as one of the greatest surgeons of the age,* after a long

* J. Rhea Barton, M. D.

sojourn in the great capitals of Europe, assured me that he felt most proud of the American profession after he had been enabled to thoroughly compare it with that of Europe. "Sir," said he, "we stand as a profession on at least an equal footing, despite the many educational advantages which they are supposed to enjoy. I returned," continued he, "with a deeper impression of the dignity and skill of the general American Faculty." This great surgeon, equally distinguished by his skill as an operator, and his genius as a discoverer, belongs to no school, is of no party in medicine, and is remarkable for his sincerity and probity of character.

Believe me. gentlemen, when I say, that after all the abuse so unjustly heaped upon our heads by American hands, the sentiments of my good and great friend, soothed not a little the wounded spirit of one, who may have many faults, but who is not wanting in reverence for his profession, and love for his country."

The discourse of Prof. Mutter is well written, and, in the main, the matter is judicious and sensible. The object of the author is to indicate to the medical student the "*requisites* of success." A *sound constitution* is the requisite first mentioned. The principle laid down, however, in the following quotation will perhaps strike some of our readers as rather too broad:—

It has been well observed by a distinguished veteran in our ranks, "that no axiom is more clearly established, or more generally admitted, than that which declares, that all that we practically are and therefore all that we can accomplish in our present state, is, for the most part, the result of our organization. If well organized, and in health, we are in a condition to be comfortable, prosperous, and useful; but if our organization be defective or unsound, the reverse is true. To our organization we are as exclusively indebted for the character and amount of our intellectual and moral qualities, as our physical; as positively so for the strength and activity of our reason and virtue, as of our muscles and joints. However paradoxical this may appear to some, or perhaps heterodoxical to others, a thorough knowledge of the physical man testifies to its truth. The brain is as truly and as obviously the organ of feeling, sentiment, and thought, as the glands are of secretion, and the muscles of motion. A large, healthy, and well formed brain, (if in proportion to the rest of the body,) therefore, gives strength of intellect and soundness of virtue to the philosopher and statesman, as certainly and directly, as large, healthy and well formed muscles and nerves give force to the arm of the smith or the leg of the dancer! The wisdom of *Ulysses* was no less the result of organization, than the swiftness of *Achilles;* and the morality of *Seneca*, equally so with the strength of *Milo.* One person differs from another in his intellect, not because his *spirit*, or his *principles* of *mind* is different, but because he differs in his organization! Nearly all that is requisite to be learned, therefore, to insure the highest improvement of the human race, is how to bestow on individuals the best organization. Carry this improvement to the highest attainable pitch and man is as perfect as he can be made, for he is now susceptible of the highest moral and intellectual cultivation."

Of the importance of health to the medical practitioner, the author speaks in terms which, however they may seem to those who have always enjoyed that inestimable blessing, will not appear exaggerated to him who, notwithstanding the disabilities and discouragements of disease, or an enfeebled constitution, is compelled to endure the labors and anxieties of professional duties, without abatement or relaxation. True is it that in none other of the walks of life are healthful activity and vigor so desirable, so indispensable as in the practice of medicine. We quote remarks on this subject :

"The old axiom, 'mens sana in corpore sano,' is full of wisdom, and if there is one among you so unfortunate as to possess a feeble constitution, let me counsel him as one who has dearly proven the misery of such a possession, to abandon the study of medicine at once, or at least until vigor and tone have been imparted to his frame. Without health, the professional life of a man is one long, dreary night of suffering and disappointment."

The writer next comments on the qualities essential to the surgeon as laid down by Celsus, viz: that he should be *young*, *adroit*, *ambidextrous*, and *without pity*. He remarks upon the fallaciousness of the last mentioned quality, and enforces the cultivation of sympathy, but, at the same time, the ability to control it.

Next, he speaks of preliminary education, a department of the subject of medical education which, in our view, is vastly more important than any other, and where reform is chiefly needed. We think Prof. M. is correct in his location of the responsibility of imperfectly educated Physicians. The difficulty is not with the schools, the schools do all that can be done in a given period of time; but they are not competent to perform the whole business of medical education. Private, as well as public instruction is necessary, and how small a proportion of medical students receive any of the advantages of the former.

It is vastly more important that checks and barriers should be placed at the commencement, than at the termination of medical pupilage, if we would succeed in shutting out of the profession those whose qualifications are defective. We concur fully in the remarks on this topic:

"I grant that as yet the education of our students is defective, and that many reach the *Doctorate* who are better qualified for the *plough;* but shall it always remain thus? Heaven forbid! But how is the evil to be remedied; does it rest with the *schools* to accomplish this mighty task? I would that I had an answer ready for the first portion of my query! It appears to me, however, that the first step in the great reform must be taken by the *country practitioners, and those who receive pupils into their offices.* If when a young gentlemen presents himself for admission, the intended preceptor would take the trouble to examine him as to his literary and scientific acquirements, and if found wanting, to assume the responsibility, and say to him, my young friend, go back for a year or two to some good literary institution, and prepare yourself for the abstruse and noble study of medicine, then come back to me and I will receive you with pleasure, much might be accomplished; for there is scarcely a youth in a thousand who would refuse the excellent advice, and by such a course the eyes of the community at large, would be opened to the difference between an educated, scientific physician, and the graduated ignoramus."

By way of encouragement to those whose opportunities for preparatory education have of necessity been limited, and in order to incite a spirit of emulation, the author adduces some of the glorious instances in which genius and perseverence have overcome every obstacle, and in which sister sciences, and even pursuits having no relationship with those of medicine, have been cultivated with distinguished success by those who, at the same time, were, or are eminent as medical practitioners. This is not the least interesting portion of the discourse, but we cannot do it justice without making larger quotations than our limits will allow.

The remaining "requisites" which the Professor enforces are, *a love for the profession, habits of industry, steadiness of purpose, good manners, good temper, and finaly, strict morality and virtue.* His remarks under each of these heads commend themselves to the judgement and taste of the reader.

In leaving the introductories of the Jefferson College, we hope we may be pardoned for expressing regret that a disposition to slur at the recommendations of the National Convention is too palpable to be overlooked. We trust that this institution may be convinced that the best interests of the profession, and its own best interests, will be subserved by a hearty concurrence, and co-operation in the objects proposed by the organization of a deliberative body representing the views and wishes of the profession of the whole country.

The only remaining introductory emanating from Philadelphia which our budget contains, is from the pen of Prof. D. Pereira Gardener, on the *relations of chemistry to the vital force*. Doct. Gardener is Prof. of Chemistry and medical jurisprudence in the fifth, (chronologically enumerated) of the medical institutions of Philadelphia. He is also favorably known as editor of a Medical Dictionary recently published.

The writer endeavors to illustrate the position that chemical and vital forces are so closely blended that, if, not identical, no line of demarcation can be drawn between them. He appears to advocate the doctrine of "equivocal generation." The views which he presents will not suit those who adhere to vitalism with a kind of religious infatuation. looking upon chemico-vitalism with as much obhorence as if it were a fatal heresy. For ourselves we are unable to see why purely vital facts should be considered more sacred than facts appertaining to physical or chemical science, inasmuch as all are equally attributable to Divine wisdom and power, and all are equally inscrutable by human comprehension. We are satisfied to seek truth, so far as our limited capacities will enable us to apprehend it, through any channel, and if by the application of physical or chemical principles to the phenomena of life, our knowledge of the latter will be extended, we can perceive no reason for dissatisfaction.

Prof. Gardener's lecture is more strictly introductory to the subjects of which he is to treat, and more philosophical in its character, than any of the discourses of which we have spoken. It is replete with striking facts and thoughts, clothed in highly figurative language. We select, almost at random, an extract which will serve as a specimen of the author's style, and of the views which he presents:

"Wherever a determinate combination of certain elements and the cellular figure are present, there the capacity to live is also. It is not in complex organs, in separated senses, in the perfection of vision, in a nice apparatus for the detection of sounds or odors, nor in members for locomotion, that life is alone manifested. These are improvements in the machinery but not in the agent, nor are they essential to the manifestation of a high degree of vitality. There may be found, during the summer, attached to the rootlets of the duckweed, little masses of a gelatinous substance ; these are the creatures called Hydræ. It will be seen that the body is a clear, apparently homogenous jelly, the form rudimentary, consisting of a stem and a small sack surrounded by a few thread-like tentacula. Touch the water in which they sport, the creatures cease to move and fold themselves into a minute spheres, conscious of danger, possessed of the instinct to avoid it. Observe how they climb the sides of the vessel by their tentacula ; how they move on a plain by a series of rapid somersets ; with what skill they swim ; how they suspend themselves from the surface of the liquid, watching for prey ; man's capacity for locomotion is nothing to that of this animated speck. See the contentions of two who have some cause of quarrel ; how they dart on each other and wrestle! Of what do they dispute? There is no apparent object of contention to justify so much strife—is it mere ill nature, the pride of strength, or a question in politics? Here are the perfection of locomotion, a capacity for enjoyment, anger, stratagem, self-preservation, fear, exquisite sensation, apparent sight, in a dot of jelly. But take a needle and break one of them into a hundred atoms, and each dilates into a new animal, vigorous and subtle as its parent. And why? Because the homogeneous cellules are each endowed with all the vitality of the hydra, which gains in its tentacula and stem only mechanical implements, not additional life. *What vital quality is here, and it is equal to the highest manifestation, is the property of every atom.* The separation of the senses into appropriate organs, adds to the convenience of the larger animals, but it cannot be shown that vitality is thereby increased. In all cases there is a nice adaptation perceptible between the organization and the wants of the being. The field of vision of the insect is but a point compared to that of man, but that minute space is ample for all his wants ; to him it is a world rich in varied interest, creatures unseen by us are there disporting, tints, inappreciable to our gross sense light up every object—*there is a new universe in that point.* A world of music, a world of sensation lie around us, full of life, but which our organization cannot appreciate. Nor is this a subject of regret, for to possess this power would debar from greater ; would reduce the body from the position assigned it as the tabernacle of the soul. Thus the poet :

"Why has not man a microscopic eye ?
For this plain reason, man is not a fly,
Say what the use, were finer optics given,
To inspect a mite, not comprehend the heaven ?

Or touch, if tremblingly alive all o'er,
To smart and agonise at every pore ?
Or quick effluvia darting through the brain,
To die of a rose in aromatic pain ?"—POPE.

So far as the properties of mineral and organic matter can be compared, there is not much dissimilitude. Living objects may not be larger than the ultimate atoms of bodies. The Rev. Mr. Reade states that forty-one thousand millions of Gaillonellæ occupy but a cubic inch, and Ehrenberg that ten thousand thousand millions of Bacillariæ are necessary to the same space, and each particle of these animals is living and characterized by the cellular figure. *Hence, it would appear that the property of life belongs to matter, the ultimate atoms of which are cellular and distinguishable from the solid spheroids of inanimate substances.* Again, if in the development of a crystal, millions of particles can be arranged in a symmetrical position during a short time, so in the multiplication of living things millions can be born in an hour. The eminent microscopist, Ehrenberg, has shown that from one almost imperceptible corpuscle, the Gaillonellæ increase in four days to 170 billions, and occupy a space of two cubic feet. Thus vital matter may be as minute as elementary atoms, as rapidly formed from mineral substances as a crystal can be deposited, may remain centuries inactive, and seems to be characterized by no peculiar physical properties. Moreover nearly if not all the elements can become the constituents of living cellules.

How then does the vital condition originate ? We are told that it is supernatural, metaphysical. Impregnate distilled water with carbonic acid, add a drop of yeast, leave a little air in the bottle, and seal, expose to sunlight in the summer, and it will be found in a few days that a rich green vegetation has made its appearance. Dissolve an organic acid, or any of its salts, and before long there will be developed a fleecy conferva. In a fermenting solution of sugar, saccharomyces originate. Vegetable infusions in a few hours swarm with animalcules. In such cases it is impossible to discover anything more than a suitable condition of matter, arising not from supernatural agency, but from a peculiar grouping of the atoms, for there can be no seeds present. But in creatures which possess a more complicated frame-work, a cellule or aggregate of cellules is the parent, and this appears to be necessary to perpetuate the mechanism. Reproduction by germs is not an essential characteristic of vitality, for hundreds of genera perpetuate their species by fissiparous generation."

Since the foregoing, was written, by the kindness of a neighbor, we have had the pleasure of perusing another Philadelphia introductory, to wit., *a biographical memoir of the late Dr. George Mc Lellan by Prof. Darrack of the Pennsylvania Medical School.* The institution with which Prof. D. is connected was founded chiefly through the instrumentality of Dr. McLellan. The subject, therefore, was extremely appropriate on the occasion of the opening of the session succeeding his decease. The biographer, moreover, had been an early friend of the deceased, and sustained relations of intimate friendship during his brief, but brilliant professional career. We cannot do justice to this production, and to the subject, by a few extracts. Presenting, as it does, a highly interesting sketch of a lamented member of our profession, distinguished for his genius, industry, and zeal, it should be read entire, and were it to be generally circulated, as we hope it will be, we doubt not it would be one of the most generally acceptable of the introductories of the season. It is an able, feeling tribute to the memory of a talented Surgeon, and a cherished friend, and redounds to the credit of both heart and head of the author.

Here we leave the subject of *introductory addresses* for the present, designing to notice the remainder in the next number.

